# Temperature Compensation for Conductivity-Based Phase Fraction Measurements with Wire-Mesh Sensors in Gas-Liquid Flows of Dilute Aqueous Solutions

**DOI:** 10.3390/s20247114

**Published:** 2020-12-11

**Authors:** Philipp Wiedemann, Felipe de Assis Dias, Eckhard Schleicher, Uwe Hampel

**Affiliations:** 1 Institute of Fluid Dynamics, Helmholtz-Zentrum Dresden-Rossendorf, Bautzner Landstraße 400, 01328 Dresden, Germany; f.dias@hzdr.de (F.d.A.D.); u.hampel@hzdr.de (U.H.); 2 Chair of Imaging Techniques in Energy and Process Engineering, Technische Universität Dresden, 01062 Dresden, Germany

**Keywords:** wire-mesh sensor, temperature compensation, multicomponent electrolyte solution, ionic conductivity, two-phase thermohydraulics

## Abstract

Wire-mesh sensors are well-established scientific instruments for measuring the spatio-temporal phase distribution of two-phase flows based on different electrical conductivities of the phases. Presently, these instruments are also applied in industrial processes and need to cope with dynamic operating conditions increasingly. However, since the quantification of phase fractions is achieved by normalizing signals with respect to a separately recorded reference measurement, the results are sensitive to temperature differences in any application. Therefore, the present study aims at proposing a method to compensate temperature effects in the data processing procedure. Firstly, a general approach is theoretically derived from the underlying measurement principle and compensation procedures for the electrical conductivity from literature models. Additionally, a novel semi-empirical model is developed on the basis of electrochemical fundamentals. Experimental investigations are performed using a single-phase water loop with adjustable fluid temperature in order to verify the theoretical approach for wire-mesh sensor applications and to compare the different compensation models by means of real data. Finally, the preferred model is used to demonstrate the effect of temperature compensation with selected sets of experimental two-phase data from a previous study. The results are discussed in detail and show that temperature effects need to be handled carefully—not merely in industrial applications, but particularly in laboratory experiments.

## 1. Introduction

Wire-mesh sensors were introduced by [1] and evolved into well-established scientific instruments for measuring the spatio-temporal phase distribution of two-phase flows. Due to the versatile measurement principle, which is based on differentiating phases by their electrical conductivities, the fields of application steadily extended from fundamental research on thermohydraulic systems related to nuclear power stations [2] across solar thermal applications [3] up to chemical engineering research [4,5]. For the purpose of measuring multiphase flows of non-conducting fluids, e.g., in the oil and gas industry, also a capacitance-based measurement principle was developed, cf. [6]. The latter one is, however, not considered in the present study. In contrast, recently developed dual-modality sensors [7,8], which combine both measurement principles in order to differentiate phases in three-phase flows of oil, water and gas, may benefit from the temperature compensation approach proposed in this article.

In the context of conductivity-based wire-mesh sensors that are further focused here, most applications are related to two-phase flows of a gas or vapor phase and liquid water. The quantification of phase fractions is commonly based on scaling the measured signals with the aid of a single-phase reference measurement of water and an appropriate normalization approach, cf. e.g., [9]. Since the electrical conductivity of water is known to be temperature-dependent [10,11,12,13], the temperatures of the single-phase reference measurement and the two-phase measurement must be identical in order to calculate correct phase fractions. Although the so-called histogram calibration offers a possible alternative, it is not universally applicable, as it fails for e.g., annular or stratified flow patterns, cf. [9].

Even though laboratory set-ups in the scientific environment provide almost ideal conditions, the temperature needs to be accounted for, e.g., if water is circulated in flow loops, as reported in [14]. Depending on the flow conditions the liquid phase may heat up due to intensive pumping or cool down in case of large gas flow rates at which latent heat of evaporation for humidifying the gas is supplied by the liquid. As long as such generic set-ups are used, appropriate reference measurements can be easily recorded by turning off the gas flow. However, this still leads to interruption of the work flow and prolongs measurement campaigns. In contrast, wire-mesh sensors are increasingly encountered in applied research and industry [3,15] and the acquisition of suitable reference measurements becomes a challenging task due to demanding operating conditions, the large-scale set-up as well as dynamic operation modes. Especially in case of vapor-liquid flows operated at saturation conditions, it is difficult to eliminate vapor formation, cf. [9]. Also Hoffmann [3] reports on this problem with regard to the start-up procedure of a solar thermal facility with direct steam generation. Therefore, a method, which is capable of mathematically compensating temperature effects in reference data, is absolutely required.

To the best of the authors’ knowledge the first and only attempt of correcting temperature effects in phase fraction measurements with wire-mesh sensors was made by Manera [16,17] in the context of investigating flashing vapor-liquid flows. She applied a first order polynomial to describe the relation of measured voltage, which is associated with the fluid’s conductivity, against the fluid’s temperature. The coefficients were determined by applying regression analysis to own experimental data within the temperature range under investigation. Although she demonstrated functionality of the linear approximation, it is generally restricted to a rather small temperature range, as the conductivity is known to feature a non-linear temperature characteristic, cf. [13]. Moreover, the used coefficients have not been published and the method cannot be used directly.

Therefore, the present study aims at providing a theoretical basis for temperature compensation, suggesting a reliable compensation model as well as demonstrating its effect in the analysis of experimental two-phase data in detail for the first time. The article is organized as follows: Starting with a recapitulation of the measurement principle of conductivity wire-mesh sensors, Section 2 subsequently presents our approach to temperature compensation with the aid of both empirical models from the literature and a novel semi-empirical model derived from electrochemical fundamentals. The experimental set-up used for validation purposes is described in Section 3. The corresponding results are presented in Section 4 and an example of application to two-phase flow is subsequently discussed. Finally, the conclusions drawn in Section 5 emphasize the need to raise the awareness for temperature effects in wire-mesh sensor measurements and show that our compensation method presents a suitable tool for handling such effects in different applications.

## 2. Theory

### 2.1. Wire-Mesh Sensor Measurement Principle

**Figure 1:**
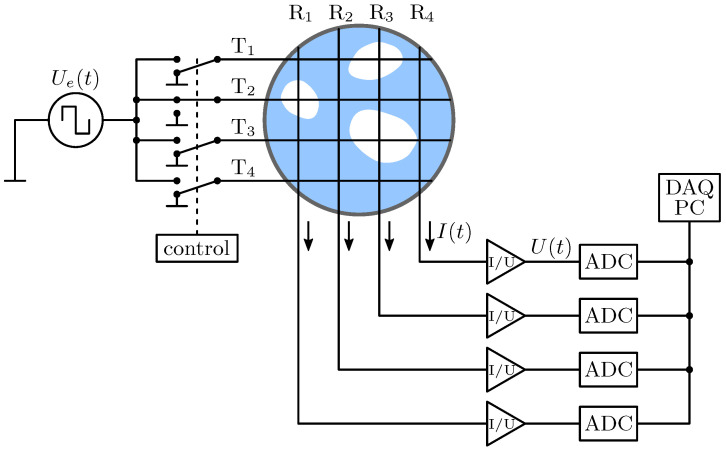
Schematic representation of the wire-mesh sensor. Adapted from [1].

A conventional wire-mesh sensor (WMS) consists of two parallel planes of parallel wires that are spanning the cross-section of a pipe. The planes are arranged perpendicularly with regard to the direction of the wires and feature a small axial distance. The wires of one plane act as transmitters, whereas the wires of the other plane operate as receivers, see $T_{i}$ and $R_{j}$ in Figure 1 respectively.

An alternating excitation signal $U_{e}\left(t\right)$ is switched to the transmitter wires consecutively. Due to the different electrical conductivities of the fluids under investigation and their spatial distribution, different conductances *G* are present at the virtual crossing points i,j of the transmitting and receiving wires. The resulting electrical currents at the receiver wires $I_{i,j}\left(t\right)$ are converted into voltage signals $U_{i,j}\left(t\right)$ by transimpedance amplifiers. Sample and hold circuits are used to extract the DC components that are subsequently digitized and sampled in parallel for all receiver wires, cf. [1]. After all transmitter wires have been activated once, a two-dimensional matrix $U_{i,j}$ is obtained for this frame *k*.

Under the assumption of ideal components being installed in the electric circuit, the measured output voltages are directly proportional to the instantaneous conductances at the virtual crossing points:

(1) $$U_{i,j,k}\;\propto \;G_{i,j,k}=\kappa_{i,j,k}\;k_{g,i,j}$$

Since the local (i,j) instantaneous (*k*) conductance is given as product of the conductivity $\kappa_{k}$, which may vary according to the presence of the fluids, and a geometry factor $k_{g}$, which is time-invariant for each individual crossing point, the measured voltage signals scale proportionally with $\kappa_{k}$ also. However, the open literature does not provide an explicitly specified range of conductivities, in which this behavior is attested. As preliminary investigations also indicated non-linearities at very high conductivities due to parasitic currents most likely, a verification of Equation (1) is required to allow for application of all further descriptions related to temperature compensation. Therefore, experimental evidence is provided in Section 4.1 for the conductivity range being investigated here.

For the application of the above measurement principle to two-phase flows of a high and a low conducting fluid with $\kappa^{H}$ and $\kappa^{L}$, respectively, local instantaneous phase fractions can be consequently quantified by normalizing the measured voltage signals with respect to single-phase reference measurements $U_{i,j}^{H}$ and $U_{i,j}^{L}$. For this purpose, several models are available for different geometrical configurations of the phases within a crossing point, cf. [9]. The simplest conversion is a linear approximation, in which the volumetric fraction of the phase with lower conductivity is calculated as

(2) $$\alpha_{i,j,k}^{L}=\frac{U_{i,j}^{H}-U_{i,j,k}^{\mathrm{meas}}}{U_{i,j}^{H}-U_{i,j}^{L}}.$$

As the present study focuses on gas-liquid flows including a non-conducting gas or vapor phase ($\kappa^{L}=0\;\Rightarrow \;U_{i,j}^{L}=0$), Equation (2) simplifies to

(3) $$\alpha_{i,j,k}^{L}=1-\frac{U_{i,j,k}^{\mathrm{meas}}}{U_{i,j}^{H}}.$$

In this context, we define $\kappa ≔\kappa^{H}$ for ease of reading in all further explanations. For flows with high dispersion levels, i.e., the interfacial structures are smaller than the spatial resolution of the sensor, e.g., in dispersed bubbly flow, other normalization approaches such as the Maxwell model are more suitable than the linear one, cf. [9,18,19]. Regardless of the model for phase fraction calculation, a single-phase reference matrix $U_{i,j}^{H}$ is needed to eliminate the influence of the geometry factors in the two-phase measurement matrix $U_{i,j,k}^{\mathrm{meas}}$. Although a few flow patterns allow for using a histogram method to determine $U_{i,j}^{H}$ from $U_{i,j,k}^{\mathrm{meas}}$ directly, cf. [9,20], recording the reference matrix separately is the method of choice in the majority of cases. With that in mind, the temporal offset between reference measurement and two-phase measurements can lead to mismatching data due to differing operating conditions and thus incorrect phase fractions. In the context of temperature changes, appropriate compensation can be provided by the method proposed in the following section. From a technical point of view only temperature measurements on the liquid phase near the wire-mesh sensor are additionally required for this purpose.

### 2.2. Temperature Compensation

#### 2.2.1. General Approach

The electrical conductivity of water is known to exhibit a strong dependence on temperature and several empirical models have been developed in the past in order to enable the conversion to a fixed reference temperature, cf. [21]. Since several decades commercially available conductivity meters apply temperature compensation for a reference temperature of $T_{ref}=25\;{}^{\circ }C$ by the generally accepted method shown in Equation (4).

(4) $$\kappa_{25{}^{\circ }C}=\frac{\kappa_{T}}{f_{25{}^{\circ }C}\left(T\right)}$$

Here, $\kappa_{T}$ refers to the actual conductivity measured at temperature *T* (in ${}^{\circ }C$). The compensation factor $f_{25{}^{\circ }C}$ is usually approximated by a first order polynomial

(5) $$f_{25{}^{\circ }C}=1+\alpha \;\cdot \;\left(T-25\;{}^{\circ }C\right),$$

in which the temperature coefficient α can be a constant or a function of *T*, see e.g., [12].

For the objective of providing a universal temperature compensation method, which is applicable to WMS measurements with any combination of *T* and $T_{ref}$, we suggest to invert the above problem and substitute the denominator $f_{25{}^{\circ }C}$ in Equation (4) by a more general compensation factor *F* that allows for both variable *T* and variable $T_{ref}$. The conductivity at temperature *T* can hence be calculated as

(6) $$\kappa_{T}=F\left(T,T_{ref}\right)\;\cdot \;\kappa_{T_{ref}}.$$

Taking into account Equation (1) the compensation factor $F(T,T_{ref})$ also applies to the local WMS signals in case of the liquid reference:

(7) $$F\left(T,T_{ref}\right)=\frac{\kappa_{T}}{\kappa_{T_{ref}}}=\frac{U_{i,j}^{H}\left(T\right)}{U_{i,j}^{H}\left(T_{ref}\right)}$$

A single-phase reference matrix $U_{i,j}^{H}$ recorded at an arbitrary temperature $T_{ref}$ can thus be converted to a reference matrix at temperature *T* corresponding to the two-phase measurement $U_{i,j,k}^{\mathrm{meas}}$. Consequently, correct phase fractions are obtained from Equation (3).

The temperature compensation factor $F(T,T_{ref})$ can be determined in different ways. In Section 2.2.2 we present a straightforward concept that allows for using $f_{25{}^{\circ }C}$ models from the literature. A novel semi-empirical approach is summarized in Section 2.2.3 and deduced in detail in Appendix A.

#### 2.2.2. Literature Models

Commonly encountered literature models are designed to provide $f_{25{}^{\circ }C}\left(T\right)$ for Equation (4). However, when being applied to both *T* and $T_{ref}$, inserting into Equation (7) yields

(8) $$F\left(T,T_{ref}\right)=\frac{f_{25{}^{\circ }C}\left(T\right)}{f_{25{}^{\circ }C}\left(T_{ref}\right)}.$$

Therefore, the compensation factor $F(T,T_{ref})$ can be derived from any $f_{25{}^{\circ }C}$ literature model. The following models are considered in the present study.

1. For natural waters (such as river or ground waters) of $\kappa_{25{}^{\circ }C}=\left(60\ldots 1000\right)\;\mu S{\mathrm{cm}}^{-1}$ the calculation of $f_{25{}^{\circ }C}$ can be accomplished according to the ISO 7888 model, cf. [22]. The corresponding result of Equation (8) is therefore denoted as $F_{\mathrm{ISO}\;7888}\left(T,T_{ref}\right)$.
2. McCleskey [12] presents a model in type of Equation (5) that particularly focuses on extending the application range of temperature compensation to acidic waters with pH<4. In this range the migration of hydrogen ions becomes increasingly dominant, because the mechanism of proton transfer among the water molecules is different from that of dissolved electrolytes that move as individual entities surrounded by a hydrate shell, cf. [10,11,12]. However, for pH>5, which is typically given for circumneutral waters in laboratory environments as well as for slightly basic water-steam-cycles of power plant applications (pH≈8.5…10 according to [13]), the influence of hydrogen ion transport vanishes and the temperature coefficient of McCleskey’s model reduces to the following equation.
  (9) $$\alpha \left(T\right)=1.85\;\times \;10^{-2}+5.37\;\times \;10^{-5}\frac{1}{{}^{\circ }C}\;\cdot \;T$$

Equation (9) is used in the resulting formulation of the compensation factor

(10) $$F_{\mathrm{McCleskey}}\left(T,T_{ref}\right)=\frac{1+\alpha \left(T\right)\;\cdot \;\left(T-25\;{}^{\circ }C\right)}{1+\alpha \left(T_{ref}\right)\;\cdot \;\left(T_{ref}-25\;{}^{\circ }C\right)}.$$

Both compensation factors $F_{\mathrm{ISO}\;7888}$ and $F_{\mathrm{McCleskey}}$ were applied to own experimental data and the results are presented in Section 4.2.2.

#### 2.2.3. Novel Semi-Empirical Model

Since the models in Section 2.2.2 relate to types of waters that differ from demineralized or deionized waters, which are mainly encountered in WMS related research and applications, we decided to develop an additional and more appropriate model. In this context, we also established a more theoretically-based approach when compared to the empirically guided models of the previous section. The derivation of our new model is comprehensively presented in Appendix A and only a brief description is given below.

The novel semi-empirical model bases on electrochemical fundamentals and describes the temperature characteristic of the electrical conductivity of a dilute aqueous solution of strong electrolytes in an analytical way. More precisely, it starts with the definition of the electrical conductivity as the sum of the ionic conductivities from all dissolved electrolytes forming the multicomponent mixture. The conductivities of the individual ions are obtained from the Debye-Hückel-Onsager (DHO) theory for binary cation-anion pairs as well as by applying the averaging procedure of Anderko and Lencka [23] in order to capture the effects of multicomponent interactions. In the course of deducing the model we assume that strong electrolytes are present exclusively and that the concentrations of the ions in the solution are constant. Eventually, we end up with the following formulation for the electrical conductivity of the solution:

(11) $$\begin{array}{c} \kappa =\frac{1}{\eta }\left[A^{*}-\frac{B^{*}}{\sqrt{\varepsilon T}}-\frac{C^{*}}{\sqrt{{\left(\varepsilon T\right)}^{3}}}\right] \end{array}$$

Here, the absolute temperature *T* (in K) as well as the dependent thermophysical properties of the solvent, i.e., its dynamic viscosity η and its permittivity $\varepsilon =\varepsilon_{r}\;\varepsilon_{0}$, represent the variables, whereas $A^{*}$, $B^{*}$ and $C^{*}$ are constants that characterize the chemical composition of the multicomponent solution.

Applying Equation (11) in Equation (7) leads to the formulation of the associated compensation factor

(12) $$F_{\mathrm{DHO}}\left(T,T_{ref}\right)=\frac{\kappa_{T}}{\kappa_{T_{ref}}}=\frac{\eta_{T_{ref}}}{\eta_{T}}\frac{g(x_{T})}{g(x_{T_{ref}})},$$

in which g(x) with x=εT is introduced to summarize the bracketed expression in Equation (11). Now Equation (12) allows for estimating the constants $A^{*}$, $B^{*}$ and $C^{*}$ from experimental data of κ(T) by means of regression analysis using selected $T_{ref}$. We applied this procedure to own experimental data that was acquired as described in following section. The resulting compensation factor $F_{\mathrm{DHO}}$ is then compared to the previously shown literature models in Section 4.2.2.

Additionally, the following approximation of Equation (12), which is also derived in Appendix A, is also discussed in Section 4.2.2.

(13) $$F_{\eta }\left(T,T_{ref}\right)=\frac{\eta_{T_{ref}}}{\eta_{T}}$$

## 3. Experiments

### 3.1. Experimental Set-Up

**Figure 2:**
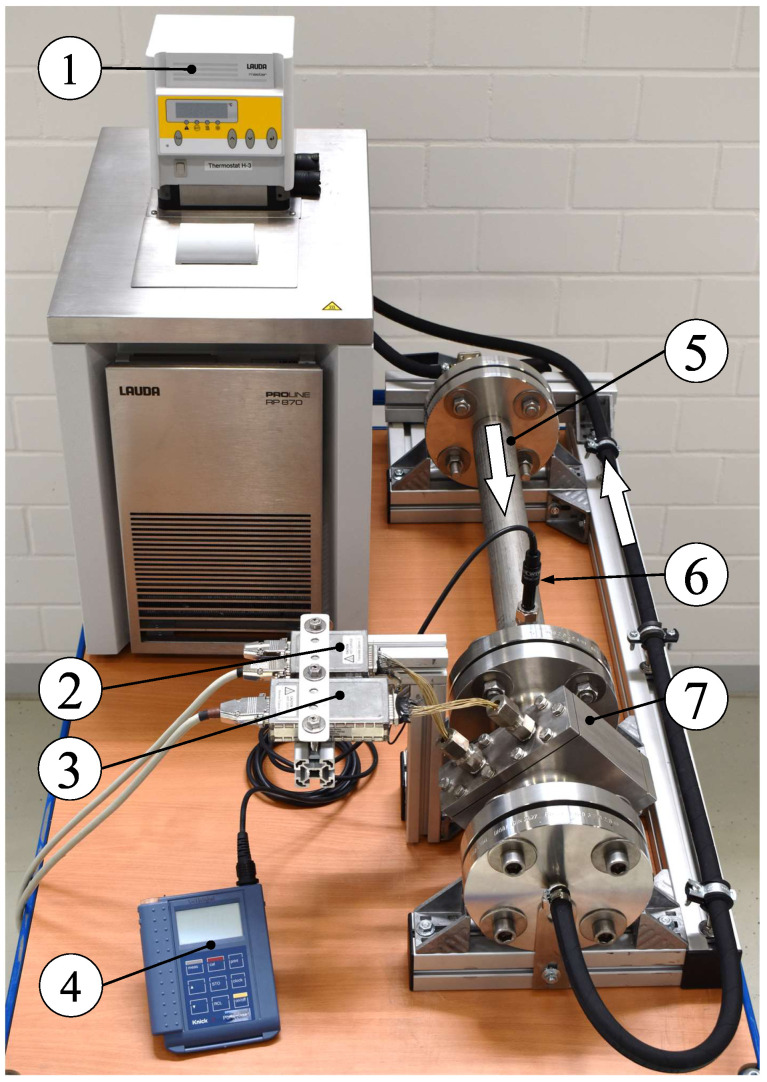
Experimental set-up comprising: (1) thermostat, (2) transmitter module, (3) receiver module, (4) measurement device for conductivity and temperature, (5) test section with (6) conductivity and temperature probe and (7) industrial wire-mesh sensor. White arrows indicate flow direction.

Experimental investigations were performed using a single-phase water loop that allows for precise adjustment of the fluid’s temperature. The set-up is depicted in Figure 2 and comprises a WMS for high temperature and high pressure applications that was installed in a test section made of a 53 mm i. d. stainless steel pipe. The test section was mounted on a frame and connected to a LAUDA ProLine RP870 thermostat via flexible tubes. For the present study a WMS with 16 wires per plane was applied resulting in 256 virtual crossing points (pixels) of which 208 represent the inner cross-section of the pipe. A combined conductivity and temperature sensor WTW TetraCon 325 was installed in the test section at 10L/D from the inlet and 4L/D in front of the WMS. Associated data was recorded with the aid of a Knick Portamess 913 Cond.

### 3.2. Experimental Procedure

Prior to all experiments the cell constant of the conductivity sensor was calibrated with $0.01\;\mathrm{mol}\;l^{-1}$ KCl solution. Moreover, the thermostat and the test section were rinsed several times by filling, circulating and emptying with deionized water.

The preparation of the first experiment began with filling the thermostat and the test section with approximately 10l of deionized water having a conductivity of $\kappa_{25{}^{\circ }C}=8\;\mu S{\mathrm{cm}}^{-1}$. Then, the facility was left at rest for five days to allow for achieving chemical equilibrium. Subsequently, a first run was performed according to the following descriptions and a second one on the following day in order to replicate the results.

At first and prior to any temperature change the amplifier settings of the WMS electronics were adjusted in order to avoid overdrive at maximum temperature, or more precisely, maximum conductivity of the water. For this purpose, the expected relative change was estimated by $F_{\eta }$, i.e., Equation (13), using the present room temperature at the beginning and the targeted maximum temperature of $80\;{}^{\circ }C$. Subsequently, each experimental run started with cooling from room temperature down to a minimum temperature of $12.5\;{}^{\circ }C$. Then, measurements with the WMS and the conductivity sensor were taken in 15 steps up to the maximum of $80\;{}^{\circ }C$. Temperature limitations were given by local freezing in the thermostat and the application range of the conductivity sensor, respectively. For each individual measurement point being associated with a temperature level the following steps were performed: (1) stabilization of temperature via the thermostat, (2) temporarily inclining the frame with the test section to approximately 45° to remove possibly entrapped gases, (3) 10s of WMS measurement with sampling frequency of 2500Hz and recording the associated temperature and conductivity data of the combined sensor.

After the runs with deionized water were finished, 0.5l were substituted by local tap water of $\kappa_{25{}^{\circ }C}=482\;\mu S{\mathrm{cm}}^{-1}$, which resulted in a conductivity of $\kappa_{25{}^{\circ }C}=37\;\mu S{\mathrm{cm}}^{-1}$ for the mixture. Then, after pausing five days again, two runs were also performed on consecutive days for the second experiment with the mixed water sample.

During all experimental runs the thermostat was constantly operated at maximum pumping power in order to provide the maximum liquid velocity in the test section. However, the velocity cannot be assumed constant during the experiments due to the temperature dependent viscosity of the fluid.

### 3.3. Measurement Uncertainty and Data Processing

Uncertainties of temperature and conductivity measurement are 0.3K and 0.5% of the measured value, respectively. With respect to the WMS measurements we additionally logged the internal temperatures of the transmitter and receiver modules as they are known to influence the resistance of the electronic components and hence variations influence the measured results. The values were almost constant during the experiments and showed minor fluctuations of less than ±1K around the mean of approximately $40\;{}^{\circ }C$. Preliminary investigations showed that a temperature drift of this magnitude leads to deviations of less than ±0.05% in the ADC count. This corresponds to ±2 counts in measured maximum of about 3830 and can thus be neglected for the present investigations.

Conversion of the acquired WMS data to 16 bit unsigned integer format was performed offline using a local PC and the WMS Data Processing Software [20]. Further data processing was subsequently accomplished using MATLAB^®^ (The Mathworks^®^, USA). In the present paper we report on the results of the second run of each experiment exclusively. Associated raw data is available as supplementary material.

## 4. Results and Discussion

### 4.1. Verification of WMS’s Linear Characteristic

Prior to applying and analyzing temperature compensation, its underlying assumption of proportional scaling of WMS output voltage with conductivity needs to be verified for the conductivity range being investigated here. For this purpose, the wire-mesh sensor data of each measurement point was temporally averaged over all N=25000 frames to ensure statistical reliability at first:

(14) $$U_{i,j}^{H}=\frac{1}{N}\sum_{k=1}^{N}U_{i,j,k}^{H}$$

Subsequently, the values of each individual wire crossing $U_{i,j}^{H}$ were plotted against the corresponding conductivity readings. To satisfy the relation of output voltage and conductivity from Equation (1) we took a linear function without offset as basis for ordinary least squares regression:

(15) $$U_{i,j}^{H}=f\left(\kappa \right)=g_{i,j}\;\cdot \;\kappa$$

Here, the linear coefficient $g_{i,j}$ can be associated with the individual gain of pixel i,j.

**Figure 3:**
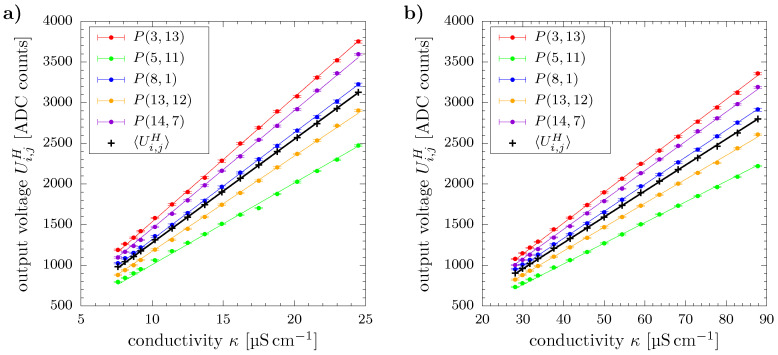
Relation of WMS output voltage and electrical conductivity of (**a**) deionized water and (**b**) water mixture for individual pixels P(i,j) (color) and cross-sectional average (black). Straight lines represent regression according to Equation (15).

For the sake of clarity, a few representative results are arbitrarily chosen from a total of 208 pixels and depicted in Figure 3. The error bars represent minima and maxima within the respective time series and indicate negligible fluctuations within the sampling period.

The overall excellent representation of the measured data by Equation (15) is also confirmed by Table 1 showing statistical characteristics of the corresponding coefficients of determination for the regression of all 208 pixels and both experimental runs. The experimental results thus verify validity of Equation (1) in the investigated conductivity range and allow for application of temperature compensation according to Equation (7).

In addition to pixel-wise data, Figure 3 and Table 1 depict the corresponding results of the cross-sectional sensor average $\langle U_{i,j}^{H}\rangle$ which is calculated as

(16) $$\langle U_{i,j}^{H}\rangle =\sum_{i}\sum_{j}a_{i,j}\;\cdot \;U_{i,j}^{H}$$

with $a_{i,j}$ denoting the share of a pixel i,j with the pipe’s cross-section, cf. [15,20]. Since excellent agreement is observed for the cross-sectional sensor average as well, it is used as representative characteristic in all further analyses.

### 4.2. Temperature Compensation

#### 4.2.1. Adjustment of the Semi-Empirical Model

In order to compare all temperature compensation models presented in Section 2.2, the constants $A^{*}$, $B^{*}$ and $C^{*}$ in our semi-empirical approach need to be determined first. For this purpose, we applied least squares regression to both experimental data sets of κ(T) using Equation (12) and selected reference temperatures of $T_{ref}=(20,40,60,80)\;{}^{\circ }C$. For the regression analysis the thermophysical properties η and $\varepsilon_{r}$ were calculated according to the formulas presented in [24], since they accurately account for density effects of the solvent. Although the formulas provide data for pure water actually, we assume applicability for dilute aqueous solutions also. The resulting constants showed similar values for all four reference temperatures and both experiments. Thus, we decided to use an uniformly averaged set finally:

$$\begin{array}{cc} A^{*} & =3.879\times 10^{-6}\;Sm\mathrm{kg}{\mathrm{mol}}^{-1}s^{-1}\\ B^{*} & =2.400\times 10^{-9}\;SK^{\frac{1}{2}}{\mathrm{mol}}^{-1}s^{-2}m^{-\frac{1}{2}}\\ C^{*} & =-1.501\times 10^{-16}\;SK^{\frac{3}{2}}{\mathrm{mol}}^{-1}s^{-4}{\mathrm{kg}}^{-\frac{1}{2}}m^{-\frac{7}{2}} \end{array}$$

Although the values of $A^{*}$, $B^{*}$ and $C^{*}$ are close to the expected order of magnitude, the negative sign of $C^{*}$ is physically not meaningful when compared to the underlying DHO theory. This indicates that the assumption of constant chemical compositions is not met in our experiments. Changing concentrations may be related to the temperature dependent dissociation of weak electrolytes [13] being possibly present in the solution and/or the temperature dependent autodissociation of water, cf. [25]. Another explanation is related to the not hermetically tight set-up that allows for interaction of the water samples and the surrounding air. Thus, concentrations of e.g., carbonate and hydrogencarbonate ions, which are linked to the carbon dioxide concentration via multiple coexisting equilibria, can be influenced by the temperature dependent solubility of the gas, cf. [26]. To reduce such effects in future studies, further investigations will be performed with closed systems using degassed water at preferably higher temperatures. Nevertheless, we consider the DHO model according to Equation (12) with the above constants in the following evaluation, as it represents the measured temperature behavior in a semi-empirical way.

#### 4.2.2. Comparison of Temperature Compensation Models

**Figure 4:**
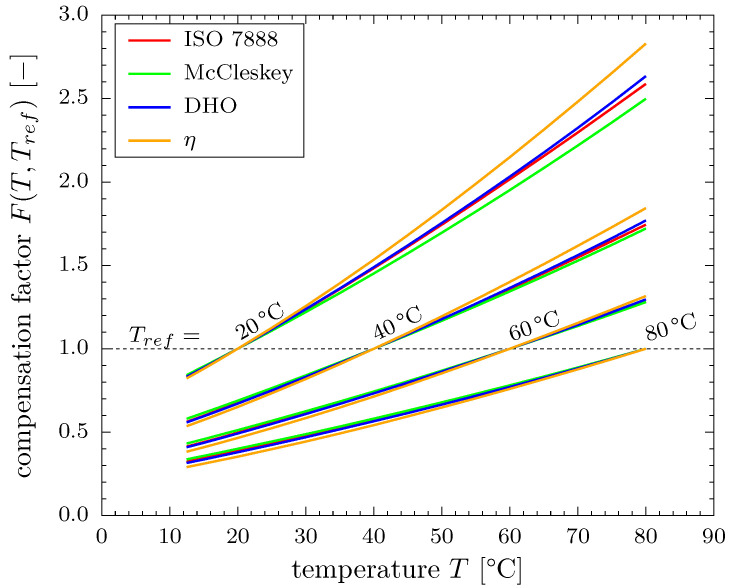
Comparison of modeled compensation factors at four different reference temperatures.

Compensation factors $F(T,T_{ref})$ were exemplarily calculated for the experimental temperature range and $T_{ref}=(20,40,60,80)\;{}^{\circ }C$ using the models from Section 2.2. The results are depicted in Figure 4 and qualitative agreement is observed among all models for varying *T* and $T_{ref}$. Deviations between each other increase with increasing distance between *T* and $T_{ref}$. All models display a significant non-linear characteristic and thus confirm that the linear approach of Manera [16,17] can be used as a first approximation within small temperature ranges only.

Figure 4 further shows that increasing gradients are observed around F=1 for smaller reference temperatures. This behavior can be attributed to the dominant influence of the dynamic viscosity that exhibits the same temperature characteristic inversely, cf. data in e.g., [24]. A fixed temperature difference will hence have a stronger influence in a typical laboratory experiment near room temperature than in an industrial high temperature application.

From a quantitative point of view, Figure 4 shows that compensation factors of $F(T,T_{ref})\approx 2.5\ldots 2.8$ result for the conversion according to Equation (7) if a reference measurement is taken at $T_{ref}=20\;{}^{\circ }C$ and should be applied to a measurement at $T=80\;{}^{\circ }C$. This means that 2.5…2.8 times higher ADC values will occur. However, even if applied to a lower temperature of $T=30\;{}^{\circ }C$ the compensation factor amounts to $F(T,T_{ref})\approx 1.25$ indicating an error of up to 25% in the measured signals if temperature compensation is neglected. Temperature compensation is therefore also important at relatively small temperature differences in practice.

**Figure 5:**
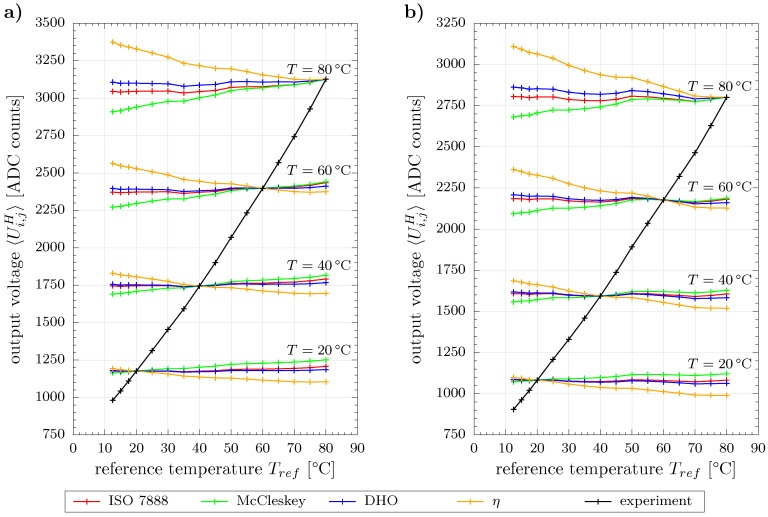
Application of temperature compensation to cross-sectional average of measured output voltages for (**a**) deionized water and (**b**) water mixture.

In order to evaluate the performance of the temperature compensation models, we applied them to the cross-sectional averages of the measured output voltages of the WMS. More precisely, the experimental data is treated as reference measurement $\langle U_{i,j}^{H}\rangle \left(T_{ref}\right)$ and compensated according to Equation (7) in order to give $\langle U_{i,j}^{H}\rangle \left(T\right)$ at four exemplary temperatures $T=(20,40,60,80)\;{}^{\circ }C$, cf. Figure 5.

A perfectly working compensation method should yield horizontal lines with this kind of representation. However, only the ISO 7888 and DHO models predict reasonable results for both liquids, deionized water and the mixed water sample. The application of the simplified η model apparently yields strong overcompensation, whereas McCleskey’s model results in a slightly underestimated compensation.

**Figure 6:**
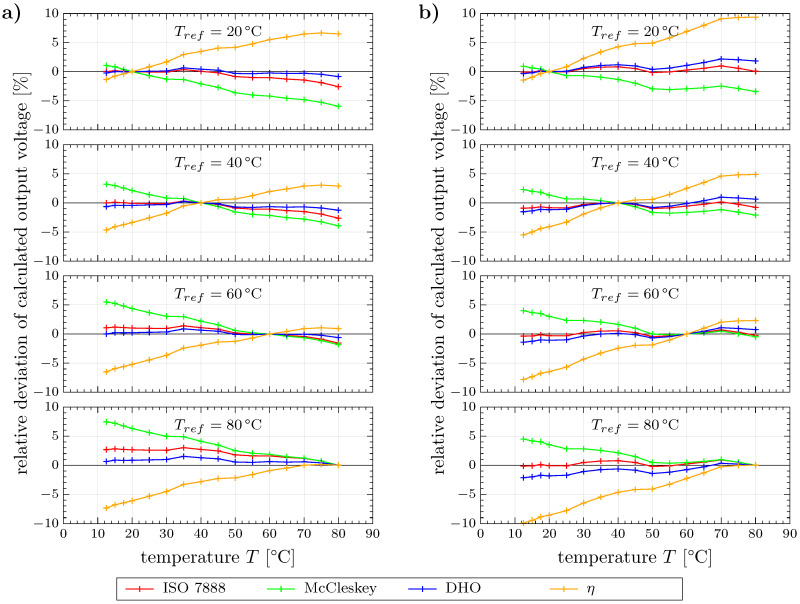
Relative deviation of compensated output voltages (cross-sectional average) for (**a**) deionized water and (**b**) water mixture.

In order to quantitatively assess the models we return to the more practical view of fixed reference temperatures, $T_{ref}=(20,40,60,80)\;{}^{\circ }C$ exemplary, as well as variable operation temperatures in the measured range $T=\left(12.5\ldots 80\right)\;{}^{\circ }C$ and depict the relative deviations between the calculated and measured output voltages in Figure 6. For the investigated parameters and both water samples all values are within a range of ±10% deviation. Thus, even the simplified η model, which shows the largest deviations, enables a rough estimation for compensating temperature effects. The model according to McCleskey performs better for the mixed water sample, but only slightly better for deionized water. Regardless of the sample almost all deviations of the McCleskey model are in the opposite direction when compared to the η model. Both the η and the McCleskey model exhibit increasing deviations with increasing distance between *T* and $T_{ref}$. The same trend is basically observed for the ISO 7888 and the DHO model also, though less pronounced and not obvious due to generally lower deviations and a non-monotonic progression. Direct comparison of these two models reveals that our DHO approach shows better agreement for deionized water, whereas the ISO 7888 model is more suitable for the mixed water sample. This result was expected due to the different fundaments of the models. More precisely, the DHO model was derived for dilute solutions particularly, whereas the ISO 7888 characterizes natural waters that are resembled by the water mixture more likely. However, the results in Figure 6a demonstrate that the validity range of the ISO 7888 model can be extended down to conductivities of $\kappa_{25{}^{\circ }C}=10\;\mu S{\mathrm{cm}}^{-1}$ with a maximum error of 3%. In contrast, the DHO model represents the behavior of the water mixture in Figure 6b with a maximum error of about 2% only.

Table 2 summarizes the data of Figure 6 with the aid of the mean absolute and the maximum deviations. It can be seen that the ISO 7888 and the DHO models yield acceptable deviations for both liquid samples individually as well as in an overall context. Therefore, these models are considered suitable for temperature compensation in WMS data analysis. With respect to the implementation, particularly into recent WMS measurement systems with almost real time data processing, the application of the ISO 7888 model is preferred due to less computational effort when compared to the DHO model, which requires time-consuming calculations of thermophysical properties or, alternatively, table lookup and interpolation operations. The choice of the ISO 7888 model for such systems can be further justified by better performance in the range of higher conductivities, which are typically encountered in industrial applications, e.g., feed water in solar thermal applications, cf. [3]. Moreover, the constants in the proposed DHO model can be different from ours and difficult to determine in practical applications. However, the new DHO model is recommended if higher accuracy is required and computing time is of minor importance, e.g., in the context of laboratory experiments.

#### 4.2.3. Effect on Results of Two-Phase Flow Measurements

In order to demonstrate the effect of (missing) temperature compensation in the analysis of two-phase flow data, we consider a typical practical scenario that accounts for further steps of data processing. More precisely, a common phase fraction cutting procedure as well as cross-sectional averaging are additionally treated here. As this leads to a superposition of multiple effects within the final results, we recapitulate the steps individually first:

1. Time-resolved and pixel-wise phase fractions $\alpha_{i,j,k}^{L}$ are calculated from a two-phase measurement $U_{i,j,k}^{\mathrm{meas}}\left(T\right)$ according to Equation (3). The reference matrix $U_{i,j}^{H}\left(T_{ref}\right)$ can be adjusted according to Equation (7) in order to match the temperature of the two-phase measurement or any other temperature. In case that the reference temperature deviates from the temperature of the two-phase flow measurement, incorrect $\alpha_{i,j,k}^{L}$ will result for those pixels that contain liquid of any amount larger than zero. If $T_{ref}<T$ negative $\alpha_{i,j,k}^{L}$ will occur for pixels that are actually filled with liquid completely.
2. The commonly applied cutting algorithm limits the phase fraction data $\alpha_{i,j,k}^{L}$ to the interval [0…1] in a pixel-wise manner, cf. [18,20]. More precisely, all values that fall below zero by application of Equation (3) are cut off while pixels that represent pure gas are not affected. Pixels at gas-liquid interfaces remain unchanged as long as their phase fractions are located in the specified interval, even though they are flawed due to temperature effects.
3. Cross-sectionally averaged phase fractions $\langle \alpha_{i,j,k}^{L}\rangle$ are calculated analogously to Equation (16) from a set of correct, incorrect and possibly cut values of $\alpha_{i,j,k}^{L}$.

Consequently, temperature effects in the resulting time series overlap with the cutting procedure. Its application is, however, affected by the flow morphology, i.e., the instantaneous amount and distribution of pixels that represent gas, liquid or the interface.

**Figure 7:**
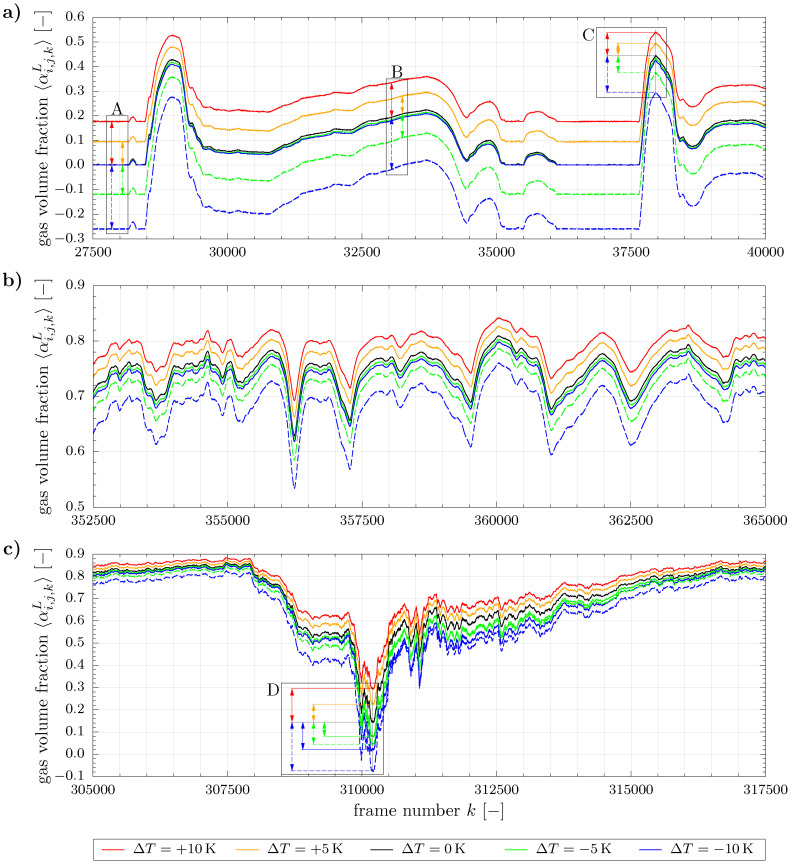
Influence of varying reference temperature on phase fraction data: (**a**) Plug flow pattern (run no. 50 in ref. [14] with $T=24.8\;{}^{\circ }C$, $v_{l,s}=0.63\;m\;S^{-1}$, $v_{g,s}=0.22\;m\;S^{-1}$), (**b**) Stratified wavy flow pattern (run no. 102 in ref. [14] with $T=26.4\;{}^{\circ }C$, $v_{l,s}=0.10\;m\;S^{-1}$, $v_{g,s}=4.80\;m\;S^{-1}$), (**c**) Slug flow pattern (run no. 106 in ref. [14], $T=25.1\;{}^{\circ }C$, $v_{l,s}=0.65\;m\;S^{-1}$, $v_{g,s}=4.79\;m\;S^{-1}$). Solid and dashed lines denote with and without application of the cutting algorithm, respectively. Details A to D are described in the text.

For the purpose of demonstrating this non-linear behavior, we exemplarily applied the above steps to selected data that was recorded in a previous study, see [14] for details. A liquid dominated plug flow pattern, a gas dominated stratified wavy flow pattern and a slug flow pattern were chosen here. Besides using the originally recorded reference matrices $U_{i,j}^{H}\left(T_{ref}\right)$, which match the temperatures of the two-phase measurements by ±0.1 K, i.e., $T_{ref}\approx T$, we also applied Equation (7) with the ISO 7888 model $F_{\mathrm{ISO}\;7888}\left(T,T_{ref}\right)$ in order to introduce artificial temperature differences to the reference matrices. For the sake of comparability, the reference temperatures were chosen to satisfy $\Delta T=T_{ref}-T=\left(+10,+5,0,-5,-10\right)\;K$, as *T* of the two-phase data varied between the measurement points. The results are depicted in Figure 7 by a couple of frames covering a representative section for each flow pattern. For a better traceability the dashed lines represent additional results for negative ΔT that were obtained without applying the cutting algorithm.

When comparing the cross-sectional gas volume fractions of positive and negative ΔT in Figure 7 to the correctly estimated series for ΔT=0K, it can be seen that higher and lower values are generally obtained, respectively. This behavior is of course related to the multiplication with the compensation factor *F* in the denominator of Equation (3). However, the extent of deviation depends on several aspects.

Firstly, when focusing on positive ΔT as well as on negative ΔT without cutting, all deviations correlate with the cross-sectional share of liquid, which is related to the magnitude of $\langle \alpha_{i,j,k}^{L}\rangle$ as a matter of course. This behavior is highlighted at three exemplary liquid levels in details A to C in Figure 7a. The strongest influence is observed for a completely liquid filled pipe (detail A) and the deviations decrease with increasing values of $\langle \alpha_{i,j,k}^{L}\rangle$ (details B and C) due to the increasing cross-sectional share of gas associated pixels, to which temperature effects do not apply. This is also confirmed by comparing the absolute differences in Figure 7a with those of any frame of the gas dominated flow in Figure 7b. The same effect is well displayed in a dynamic representation in the ascending part in Figure 7c that reflects the falling liquid level after a slug passage.

Furthermore, all arrays of curves and especially the marked details in Figure 7a reveal that larger deviations are generally detected for negative ΔT without cutting than for positive ones. The reason is given by the non-linear temperature characteristic of the electrical conductivity, as discussed earlier in the context of Figure 4 and also shown by the experimental data in Figure 5.

When incorporating the cutting algorithm for negative ΔT now, an overall reasonable approximation of the correct $\langle \alpha_{i,j,k}^{L}\rangle$ is observed in Figure 7a,b, although temperature effects are present. This is due to limiting the range of phase fractions whereby temperature related deviations are automatically corrected for all completely liquid filled pixels, as especially seen for the correctly recovered liquid reference in detail A. The remaining deviations with respect to ΔT=0K at higher $\langle \alpha_{i,j,k}^{L}\rangle$ pertain to a few pixels that represent the gas-liquid interface. In contrast, the correction effect of the cutting procedure does not apply in case of positive ΔT. Therefore, large deviations and partially physically wrong results can be obtained here, if temperature compensation is ignored.

A last essential aspect is related to the flow structure. While the inclusion of temperature compensation leads to reliable phase fractions in all flow situations, the correction effect of using the cutting procedure exclusively yields satisfactory results for flows with low interfacial area only. This can be seen when comparing the clearly separated flow structures from Figure 7a,b, in which the interfaces are well-resolved by the WMS, to the flow pattern in Figure 7c. The latter one comprises a highly aerated slug, i.e., small bubbles are entrapped in the liquid phase and produce large regions of interface, to which cutting is not applied. Especially for the frames around the minimum $\langle \alpha_{i,j,k}^{L}\rangle$ (see detail D) as well as for the subsequent section of the tail large deviations are still observed for the green and blue solid lines from the reference ΔT=0K. In contrast, considerably smaller deviations occur at comparable $\langle \alpha_{i,j,k}^{L}\rangle$ in Figure 7a,b.

Finally, Table 3 displays temporal averages of the complete 60s phase fraction time series as well as the relative errors with regard to the correctly estimated one at ΔT=0K. In accordance with the above discussion extremely large deviations are observed for the liquid dominated flow and positive ΔT. Although comparatively small deviations are obtained in the other cases due to the cutting procedure and/or the gas dominated flows, they still represent a large contribution to measurement uncertainty when compared to the analysis in [9]. Moreover, care needs to be taken when dealing with other statistical characteristics of the time series, e.g., standard deviation, skewness or span, as e.g., done in [14], since they can be influenced by temperature effects also.

In summary, the application of the proposed temperature compensation method is highly recommended in the process of quantifying phase fractions from WMS measurements. In doing so, reliable results can be obtained independently of the flow pattern and further steps of data processing. Nevertheless, under certain conditions the cutting procedure presents a simple alternative that is very likely available in most post-processing algorithms of researchers in this field. However, recent findings [9,18,19] emphasize the need for accounting for negative void fractions from an electrical point of view and advise against using the general cutting procedure. However, since negative void fractions can result from temperature effects also, temperature compensation becomes extremely important.

## 5. Conclusions

Phase fraction data from conductivity-based WMS measurements is sensitive to temperature differences between reference and two-phase measurement. The present article provides a comprehensive theoretical treatment of this effect by linking the temperature characteristic of the water’s electrical conductivity to the output signals of wire-mesh sensors. In this context, a general approach for compensating temperature effects is suggested. In addition to the potential inclusion of literature models, a novel semi-empirical model was proposed for dilute aqueous solutions on the basis of electrochemical theory.

Experimental investigations confirmed the linear characteristic of the WMS’s measurement principle up to $\kappa \approx 90\;\mu S{\mathrm{cm}}^{-1}$ and thus provided the necessary prerequisite for applying the proposed temperature compensation method. We further showed that the new model as well as the ISO 7888 model are capable of accurately compensating temperature drifts in the full range being investigated here. Our new model provides the highest accuracy when using deionized water and is therefore beneficial in laboratory applications. Due to its advantages with respect to implementation the ISO 7888 model is recommended for industrial applications.

The final demonstration of the influence of temperature effects on two-phase data revealed that a careful handling of temperature is needed—not only for dynamic operating conditions in industry, but also in laboratory experiments. Even if temperature compensation is applied, we still recommend to perform the single-phase reference measurement at a temperature being as close as possible to the temperature of two-phase measurement in order to achieve maximum accuracy.

Future work will focus on validating the proposed compensation method in a broader and industry-related operating range. For this purpose, the limitations of the WMS’s measurement principle need to be studied with respect to higher conductivities and temperatures. Beyond that, more sophisticated approaches are still required for applications, in which temperature effects are superimposed by dynamic changes in the chemical composition of the liquid phase.

## Figures and Tables

**Table 1:** Coefficient of determination.

Experiment	All Pixels			Sensor Average
	max.	min.	mean	
Deionized water	0.9999	0.9753	0.9985	0.9999
Water mixture	0.9999	0.9725	0.9984	0.9998

**Table 2:** Mean and maximum relative deviation of compensated cross-sectional sensor average.

Model	Deionized Water		Water Mixture		Both	
	mean	max.	mean	max.	mean	max.
ISO 7888	1.1%	+3.0%	0.4%	+1.0%	0.7%	+3.0%
McCleskey	2.7%	+7.5%	1.7%	+4.5%	2.2%	+7.5%
DHO	0.5%	+1.5%	0.8%	+2.2%	0.7%	+2.2%
η	2.9%	−7.4%	4.0%	−9.9%	3.4%	−9.9%

**Table 3:** Mean gas volume fractions of the analyzed gas-liquid flows and their relative deviations with respect to the temperature difference of the reference measurement.

Flow Pattern	Mean Gas Volume Fraction					Relative Deviation			
	+10 K	+5 K	0 K	−5 K	−10 K	+10 K	+5 K	−5 K	−10 K
Plug (50)	0.278	0.206	0.125	0.119	0.116	+122%	+65%	−5.0%	−7.9%
Stratified (102)	0.783	0.762	0.738	0.731	0.726	+6.0%	+3.2%	−1.0%	−1.7%
Slug (106)	0.747	0.722	0.694	0.673	0.655	+7.5%	+3.9%	−3.1%	−5.7%

## Supplementary Materials

Raw data of the single-phase experiments described in Section 3 is available online at doi:10.14278/rodare.556.

## Abbreviations

ADC	analog to digital converter
DAQ	data acquisition
DHO	Debye-Hückel-Onsager
WMS	wire-mesh sensor

## Appendix A. Derivation of the Semi-Empirical Model

According to Anderko and Lencka [23] the electrical conductivity κ of a multicomponent aqueous solution can generally be treated as

(A1) $$\kappa =\sum_{m}c_{m}\;z_{m}\;\lambda_{m}$$

with $c_{m}$, $z_{m}$ and $\lambda_{m}$ denoting the molar concentration, the absolute value of the charge number and the molar conductivity of the *m*th ion, respectively.

In a first assumption we further focus on dilute aqueous solutions of strong, i.e., fully dissociated, electrolytes only. The contribution of weak electrolytes that exhibit a concentration and temperature dependent degree of dissociation is neglected. For a single dissolved (strong) electrolyte, i.e., a binary pair of cation and anion, m=(+,−) and $\lambda_{+}$ and $\lambda_{-}$ can be obtained from the electrolyte’s molar conductivity Λ, cf. [10,11]:

(A2) $$\lambda_{\pm }=\frac{t_{\pm }\Lambda }{v_{\pm }}$$

Here, $v_{\pm }$ and $t_{\pm }$ are the multiplicity, i.e., the number of cations and anions per formula unit of the electrolyte, and the transport number of the ions, respectively. Since the transport numbers are only marginally dependent on concentration for very dilute solutions ($c\leq 0.01\mathrm{mol}\;l^{-1}$), they can be approximated by those in the limit of zero concentration $t_{\pm }^{0}$, cf. [11]:

(A3) $$t_{\pm }\;\approx \;t_{\pm }^{0}=\frac{z_{\pm }\;v_{\pm }\;\mu_{\pm }}{z_{+}\;v_{+}\;\mu_{+}+z_{-}\;v_{-}\;\mu_{-}}$$

When incorporating the ionic mobilities

(A4) $$\mu_{\pm }=\frac{z_{\pm }\;e}{6\pi \;R_{\pm }\;\eta },$$

in which $R_{\pm }$ represent the effective Stokes radii of the hydrated ions and η the dynamic viscosity of the solution, it gets clear that $t_{\pm }^{0}$ depend on geometrical and charge aspects only.

A well-established description of the molar conductivity of electrolytes, Λ in Equation (A2), is provided by the Debye-Hückel-Onsager (DHO) theory, cf. [10,11,27]:

(A5) $$\Lambda =\Lambda^{0}-\left[\alpha \left(z_{+}\;+z_{-}\right)+\beta \omega \Lambda^{0}\right]K$$

Here, $\Lambda^{0}$ represents the limiting molar conductivity at infinite dilution, i.e., zero concentration at which the ions do not interact with each other. Its formulation is given by Kohlrausch’s law of independent migration of ions, cf. [10]:

(A6) $$\Lambda^{0}=v_{+}\;\lambda_{+}^{0}+v_{-}\;\lambda_{-}^{0}=\left(v_{+}\;z_{+}\;\mu_{+}+v_{-}\;z_{-}\;\mu_{-}\right)F$$

Further terms in Equation (A5) are

(A7) $$\begin{array}{c} \alpha =\frac{N_{A}\;e^{2}}{6\pi \eta }, \end{array}$$

(A8) $$\begin{array}{c} \beta =\frac{1}{3}\frac{e^{2}}{\varepsilon_{r}\;\varepsilon_{0}\;k_{B}\;T}, \end{array}$$

(A9) $$\begin{array}{c} \omega =z_{+}\;z_{-}\frac{p\;q}{1+\sqrt{q}}. \end{array}$$

The factor *p* in Equation (A9) has been introduced in [27] to account for electrolytes of all valence families and is calculated from $z_{\pm }$, $v_{\pm }$ and $t_{\pm }^{0}$. In addition, *q* is defined as [11,27]

(A10) $$q=\frac{z_{+}\;z_{-}}{z_{+}+z_{-}}\;\frac{\lambda_{+}^{0}+\lambda_{-}^{0}}{z_{-}\;\lambda_{+}^{0}+z_{+}\;\lambda_{-}^{0}},$$

and K is the reciprocal of the Debye length

(A11) $$K=\sqrt{\frac{2\;e^{2}\;N_{A}}{\varepsilon_{r}\;\varepsilon_{0}\;k_{B}\;T}I},$$

in which *I* denotes the ionic strength

(A12) $$I=\frac{1}{2}\sum_{\pm }z_{\pm }^{2}c_{\pm }.$$

After inserting the above equations into Equation (A5), we can express the temperature dependence of the molar conductivity analytically:

(A13) $$\Lambda =\frac{1}{\eta }\left[A-\frac{B}{\sqrt{\varepsilon T}}-\frac{C}{\sqrt{{\left(\varepsilon T\right)}^{3}}}\right]$$

Here, the absolute temperature *T* (in K) as well as the dependent thermophysical properties of the solvent, η and $\varepsilon =\varepsilon_{r}\;\varepsilon_{0}$, represent the variables, whereas *A*, *B* and *C* are constants that characterize the dissolved electrolyte.

Merging Equations (A1), (A2) and (A13) consequently yields an equivalent expression for the specific electrical conductivity:

(A14) $$\begin{array}{cc} \kappa & =\underset{D}{\underset{︸}{\left(\frac{c_{+}\;z_{+}}{v_{+}}t_{+}^{0}+\frac{c_{-}\;z_{-}}{v_{-}}t_{-}^{0}\right)}}\frac{1}{\eta }\left[A-\frac{B}{\sqrt{\varepsilon T}}-\frac{C}{\sqrt{{\left(\varepsilon T\right)}^{3}}}\right] \end{array}$$

(A15) $$\begin{array}{cc} & =\frac{1}{\eta }\left[A^{\prime }-\frac{B^{\prime }}{\sqrt{\varepsilon T}}-\frac{C^{\prime }}{\sqrt{{\left(\varepsilon T\right)}^{3}}}\right] \end{array}$$

Equations (A13)–(A15) are valid for the considered binary cation-anion system, provided that the concentration of the dissolved electrolyte is sufficiently small and does not change with time or temperature, i.e., no species conversion, source or sink. If the electrolyte and its concentration were known, κ could be calculated from the above equations directly. However, typical WMS applications feature aqueous solutions of more than one electrolyte and the chemical composition is usually not known. Thus, we return to Equation (A1) and allow for a variety of cations and anions now. Since the DHO treatment covers binary cation-anion systems only and does not represent multicomponent solutions directly, we take advantage of the averaging procedure proposed by Anderko and Lencka [23]. They suggested to account for interactions of many ions with approximating $\lambda_{m}$ in Equation (A1) by an averaged one that accounts for all binary interactions:

(A16) $$\lambda_{m}\;\approx \;\bar{\lambda_{m}}=\sum_{n}f_{n}\lambda_{m(n)}$$

Here, $\lambda_{m(n)}$ represents the molar conductivity of ion *m* in presence of any counterpart *n*. More precisely, if *m* denotes a cation, *n* represents each single anion in the mixture and vice versa. According to [23] $\lambda_{m(n)}$ needs to be calculated using modified concentrations that account for the ionic strength of the multicomponent mixture $I_{mix}$:

(A17) $$c_{m}=\frac{2\;I_{mix}}{z_{m}\left(z_{m}+z_{n}\right)}\;c_{n}=\frac{2\;I_{mix}}{z_{n}\left(z_{m}+z_{n}\right)}$$

In contrast to [23], our approach makes use of the DHO theory, since it is assumed to be sufficient for dilute solutions being investigated here. Hence, $\lambda_{m(n)}$ is obtained from Equations (A2) and (A5) and the modified concentrations $c_{m}$ and $c_{n}$ are used in Equation (A12).

The fraction $f_{n}$ in Equation (A16) is given as

(A18) $$f_{n}=\frac{z_{n}\;c_{n}}{c_{eq}}$$

with $c_{eq}$ denoting the sum of $z_{m}\;c_{m}$ or $z_{n}\;c_{n}$ over all cations or anions, respectively, cf. [23].

When applying Equation (A16) in Equation (A1) along with the above modifications for multicomponent solutions and resuming the assumption of constant concentrations, we obtain the same temperature behavior as for single electrolytes in Equation (A15):

(A19) $$\begin{array}{cc} \kappa & =\sum_{m}\left(c_{m}\;z_{m}\;\sum_{n}\frac{z_{n}\;c_{n}}{c_{eq}}\frac{t_{m}^{0}}{v_{m}}\frac{1}{\eta }\left[A_{m(n)}-\frac{B_{m(n)}}{\sqrt{\varepsilon T}}-\frac{C_{m(n)}}{\sqrt{{\left(\varepsilon T\right)}^{3}}}\right]\right) \end{array}$$

(A20) $$\begin{array}{cc} & =\frac{1}{\eta }\;\sum_{m}\sum_{n}\underset{D_{m(n)}}{\underset{︸}{c_{m}\;z_{m}\frac{z_{n}\;c_{n}}{c_{eq}}\frac{t_{m}^{0}}{v_{m}}}}\left[A_{m(n)}-\frac{B_{m(n)}}{\sqrt{\varepsilon T}}-\frac{C_{m(n)}}{\sqrt{{\left(\varepsilon T\right)}^{3}}}\right] \end{array}$$

(A21) $$\begin{array}{cc} & =\frac{1}{\eta }\left[\sum_{m}\sum_{n}A_{m(n)}^{\prime }-\frac{\sum_{m}\sum_{n}B_{m(n)}^{\prime }}{\sqrt{\varepsilon T}}-\frac{\sum_{m}\sum_{n}C_{m(n)}^{\prime }}{\sqrt{{\left(\varepsilon T\right)}^{3}}}\right] \end{array}$$

(A22) $$\begin{array}{cc} & =\frac{1}{\eta }\left[A^{*}-\frac{B^{*}}{\sqrt{\varepsilon T}}-\frac{C^{*}}{\sqrt{{\left(\varepsilon T\right)}^{3}}}\right] \end{array}$$

Here, *T*, η and ε represent the variables again, whereas $A^{*}$, $B^{*}$ and $C^{*}$ are constants that characterize the chemical composition of the multicomponent solution.

Denominating the bracketed expression in Equation (A22)

(A23) $$g\left(x\right)=A^{*}-\frac{B^{*}}{\sqrt{x}}-\frac{C^{*}}{\sqrt{x^{3}}}$$

with x=εT allows for a compact formulation of a compensation factor according to Equation (7):

(A24) $$F_{\mathrm{DHO}}\left(T,T_{ref}\right)=\frac{\kappa_{T}}{\kappa_{T_{ref}}}=\frac{\eta_{T_{ref}}}{\eta_{T}}\frac{g(x_{T})}{g(x_{T_{ref}})}$$

For a solution of unknown chemical composition $A^{*}$, $B^{*}$ and $C^{*}$ can be obtained from experimental data of κ(T) using regression analysis and selected $T_{ref}$, as described in Section 4.2.1.

Additionally, a rough approximation of Equation (A24), which neglects the contribution of g(x) and uses the viscosity ratio exclusively

(A25) $$F_{\eta }\left(T,T_{ref}\right)=\frac{\eta_{T_{ref}}}{\eta_{T}},$$

is further treated here. This compensation factor $F_{\eta }$ can be associated with the temperature induced change of the ionic mobility, see Equation (A4), and thus the behavior in the limit of zero concentration, cf. Equation (A6).
